# Retraction Note: Hybrid CNN-LSTM model with efficient hyperparameter tuning for prediction of Parkinson’s disease

**DOI:** 10.1038/s41598-024-78418-y

**Published:** 2024-11-07

**Authors:** Umesh Kumar Lilhore, Surjeet Dalal, Neetu Faujdar, Martin Margala, Prasun Chakrabarti, Tulika Chakrabarti, Sarita Simaiya, Pawan Kumar, Pugazhenthan Thangaraju, Hemasri Velmurugan

**Affiliations:** 1 Department of Computer Science and Engineering, Chandigarh University, Chandigarh, Punjab India; 2 Amity School of Engineering and Technology, Amity University Haryana, Gurugram, India; 3 Department of Computer Engineering and Application, GLA University, Mathura, Uttar Pradesh India; 4 School of Computing and Informatics, University of Louisiana at Lafayette, Lafayette, USA; 5 Department of Computer Science and Engineering, Sir Padampat Singhania University, Udaipur, 313601 Rajasthan India; 6 Sir Padampat Singhania University, Udaipur, Rajasthan India; 7 Apex Institute of Technology, Department of Computer Science and Engineering, Chandigarh University, Mohali, Punjab India; 8 College of Computing Sciences & IT, Teerthanker Mahaveer University, Moradabad, Uttar Pradesh India; 9 Department of Pharmacology, All India Institute of Medical Sciences, Raipur, Chhattisgarh India

Retraction of: *Scientific Reports* 10.1038/s41598-023-41314-y, published online 05 September 2023

The Editors have retracted this Article due to concerns regarding the validity and veracity of the work presented. Specifically:

- Literature Review section partially overlaps with a previously published article^1^;
- Figures 9 and 20 were found to overlap with figures from previous publications^2,3^;
- Multiple sections of the Article contain references irrelevant to the content they are cited to support, specifically references 5, 6, 7, and 10 in the original Article;
- The Article contained multiple unusual and non-standard phrasing for standard scientific terms;
- Further analysis of the data presented in the Article found that the F-score shown in Table 5 appears to be incorrectly calculated;
- Concerns were raised regarding the Mel-spectrogram extraction. The dataset used by the authors only includes extracted features as text and numerical data, and the authors did not provide any further raw data in form of audio-files that could have been used for Mel-spectrogram extraction.

Due to these concerns, the Editors no longer have confidence in the work presented in this Article.

Pugazhenthan Thangaraju, Umesh Kumar Lilhore, Prasun Chakrabarti, and Surjeet Dalal do not agree to this retraction. Neetu Faujdar, Martin Margala, Tulika Chakrabarti, Sarita Simaiya, Pawan Kumar, and Hemasri Velmurugan have not responded to correspondence from the Editors about this retraction.
